# Integrating physical fitness and psychology: an in-depth discussion of exercise interventions for weight reduction among university students

**DOI:** 10.3389/fpsyg.2026.1836772

**Published:** 2026-06-30

**Authors:** Feng Dong, Dali Sun

**Affiliations:** 1 Department of Physical Education, Xi'an University of Technology, Xi'an, China; 2 College of Physical Education, Jilin University, Changchun, China

**Keywords:** autonomy, campus health policy, competence, exercise intervention, multi-level intervention, perceived physical literacy, physical fitness, physical literacy

## Abstract

This in-depth study challenges a prevailing assumption in the field of university student weight management that the principal lever is the prescription of ever-more-efficient exercise modalities and argues instead that the more decisive determinant is whether existing modalities are delivered in ways that satisfy students’ basic psychological needs for autonomy, competence, and relatedness, as articulated by Self-Determination Theory (SDT). We adopt SDT as the unifying theoretical lens through which we re-interpret the recurring barriers documented in the post-pandemic, smartphone-saturated university literature: weight-related self-stigma (a threat to competence and relatedness), low exercise self-efficacy (a threat to competence), academic time pressure and digital over-load (threats to autonomy), and the bidirectional load of stress, anxiety, and impaired sleep. Within this framework, structured aerobic training, high-intensity interval and sprint training (HIIT/SIT), and muscle-strengthening exercise (MSE) are re-evaluated not as competing physiological prescriptions but as need-supportive options whose suitability depends on the student’s dominant psychological barrier and profile, with explicit psychological indications offered for each. We elevate Physical Literacy (PL) the integrated motivation, confidence, competence, knowledge, and understanding required to sustain physical activity across life to a core, life-course-oriented outcome of intervention rather than a peripheral construct. The review then connects these individual-level mechanisms to multi-level institutional levers (technology-mediated coaching, environmental redesign, and policy) that are uniquely available to universities. Limitations of the underlying evidence including the predominance of cross-sectional designs, single-study findings, measurement heterogeneity, and geographic skew toward Chinese and Gulf samples are explicitly bounded. We propose a coherent, theoretically grounded, and operationally actionable framework for transforming the university stage from a high-risk window for weight gain into a launchpad for life-long Physical Literacy and wellbeing.

## 1 Introduction

The transition to university represents a pivotal developmental period characterized by a confluence of significant academic, social, and environmental shifts. Students navigate newfound independence, intensified academic pressures, altered sleep patterns, and frequently, a deterioration in dietary quality and physical activity habits (Yan et al., 2024; Yu and Ueda, 2025). This “perfect storm” of lifestyle changes creates a high-risk environment for negative health outcomes, with weight gain and the development of obesity being particularly prevalent concerns. Empirical evidence underscores the severity of this public health issue; for instance, a study of college students in Oman revealed that over 27% of the sampled population was classified as either overweight or obese. More alarmingly, this obesity was significantly correlated with tangible real-world consequences, including an associated 6.6% decrease in academic grades and a markedly higher likelihood 5.77 times greater of developing obesity-related comorbidities compared to normal-weight peers (Mondal et al., 2025). This data illustrates that the impact of student obesity extends far beyond physical health metrics, directly impairing educational attainment and long-term well-being, thereby presenting a critical challenge for higher education institutions.

Compounding the challenge of actual weight status is the frequent misalignment between how students perceive their bodies and their objective clinical classification. This discordance between self-perceived body weight and actual weight status, as determined by Body Mass Index (BMI), acts as a profound psychological barrier to initiating health-promoting behaviors. Research utilizing large, representative samples, such as the 2018 Galician Risk Behavior Data System (*n* = 7,853), found only moderate concordance between perception and reality, with a weighted Cohen’s kappa coefficient of 0.503. The study further identified important demographic variations; concordance was highest among women and differed across age groups for specific BMI categories (Blanco-Ferreiro et al., 2024). This perceptual gap means that a student who is objectively overweight or obese but perceives their weight as “normal” may lack the essential cognitive trigger the recognition of a problem required to seek change. Conversely, normal-weight students who inaccurately perceive themselves as overweight may engage in unnecessary or unhealthy restrictive behaviors (Vartanian and Porter, 2016), a discordance that has recently been re-documented in college populations using both perceived and objectively measured health metrics (Blouin et al., 2025). Therefore, the university weight management challenge is not merely physiological but is deeply embedded in a complex biopsychosocial landscape where psychological factors like body image, self-awareness, and internalized stigma interact with social environments and biological predispositions. Consequently, traditional interventions that focus solely on prescribing generic exercise regimens or nutritional guidelines are often inadequate. A student struggling with weight-related self-stigma, academic stress, poor sleep, and excessive smartphone use requires a more nuanced approach. Effective strategies must be integrative, simultaneously targeting the physiological drivers of weight gain while dismantling the psychological and behavioral barriers that sustain sedentary lifestyles and poor dietary choices. A construct that recurs throughout this review and that we identify early to flag its central role is Physical Literacy (PL). Following Whitehead (2010) and the evidence-informed conceptual model of Cairney et al. (2019), PL is defined as the integrated motivation, confidence, physical competence, knowledge, and understanding required to value and take responsibility for engagement in physical activity throughout life. Its self-report variant, Perceived Physical Literacy (PPL), is the student’s own assessment of these attributes. PL/PPL is not a parallel framework competing with SDT but a downstream, life-course outcome of the same need-satisfaction processes: autonomy provides the volitional core (“I value moving for my own reasons”), competence provides the embodied capability (“I have the skill to act on that value”), and relatedness provides the social ecology in which both can be sustained. Treating PL/PPL as a target outcome not just a means re-frames the goal of university interventions from short-term weight loss to the cultivation of the enduring motivational and physical resources that students carry into adult life.

Despite the clear public-health significance of this problem, prior reviews have addressed it only in fragments. Teixeira et al. (2012) provided an authoritative systematic review of self-determination theory and exercise, but their corpus comprised mixed adult populations and predates the post-pandemic, smartphone-saturated student environment in which contemporary motivation must be understood. Vartanian and Porter (2016) offered a careful synthesis of weight stigma and eating behavior, yet their analysis was confined to eating outcomes and did not link weight stigma to specific exercise modalities or to institutional intervention design. Cairney et al. (2019) advanced an evidence-informed conceptual model of physical literacy, but the model is generic across the life course and is not anchored in the distinctive constraints of the university context (academic time poverty, digital over-load, peer-driven social norms, and post-COVID-19 fitness erosion). Three thematic blind spots therefore persist: (i) no review has placed weight-related self-stigma, exercise self-efficacy, mental-health load, and digital saturation inside a single, theoretically unified motivational architecture for the university stage; (ii) no review has evaluated the major exercise modalities (aerobic, HIIT/SIT, muscle-strengthening) by the criterion of how well their typical delivery supports or thwarts the SDT basic needs of autonomy, competence, and relatedness in this population; and (iii) no review has connected this individual-level analysis to the institutional and policy levers that universities uniquely among adult settings actually control.

The present review is designed to address these gaps. Its specific contributions are four-fold. First, we explicitly adopt SDT as the unifying lens (Section 3.1) and re-interpret the recurring psychological barriers as patterned threats to autonomy, competence, and relatedness. Second, we re-evaluate aerobic exercise, HIIT/SIT, and muscle-strengthening exercise not as competing physiological prescriptions but as need-supportive options whose suitability depends on the student’s psychological profile (Section 6). Third, we elevate Physical Literacy from a peripheral construct to a central, sustainable end-point of intervention, integrated with rather than parallel to the SDT framework (Sections 3.1 and 7). Fourth, we map these individual-level mechanisms onto a multi-level intervention architecture (technology, environment, policy) that is feasible within the university’s institutional reach (Section 7). By doing so, this review aims to provide a coherent, theoretically grounded, and operationally actionable framework that prior partial reviews have not.

## 2 Methods

### 2.1 Review design and rationale

This article is presented as a narrative review. A narrative review was selected over a systematic review or meta-analysis because the central question is integrative and theoretical: how do physiological exercise effects, psychological barriers, contextual lifestyle factors, and institutional policies jointly shape weight-management outcomes among university students? This question spans heterogeneous designs (randomized crossover trials, longitudinal cohorts, cross-sectional surveys, validation studies, and interventions of differing duration and modality) and outcomes (BMI, body composition, fitness markers, motivation, self-stigma, sleep, mental health). A narrative synthesis is well suited to this kind of integrative interpretation across paradigms, whereas a systematic review would force premature exclusion of evidence that is essential for conceptual coherence (Greenhalgh et al., 2018; Sukhera, 2022). We have nonetheless drawn on principles of transparency from systematic reviewing explicit search strategy, defined time frame, and stated inclusion/exclusion criteria to maximize the rigor of the synthesis.

### 2.2 Search strategy

Three bibliographic databases were searched: PubMed/MEDLINE, Web of Science Core Collection, and Scopus. The search was supplemented by hand-searching the reference lists of included articles and forward-citation searching for highly relevant reports. Search terms were combined using Boolean operators within three conceptual blocks. Block 1 (population): “university student*” OR “college student*” OR “undergraduate*” OR “higher education.” Block 2 (exposure): “exercise” OR “physical activity” OR “aerobic” OR “high-intensity interval training” OR “HIIT” OR “sprint interval training” OR “resistance training” OR “muscle-strengthening exercise.” Block 3 (outcomes / psychological constructs): “weight” OR “body mass index” OR “BMI” OR “obesity” OR “body composition” OR “self-efficacy” OR “motivation” OR “self-determination” OR “weight stigma” OR “physical literacy” OR “mental health” OR “sleep.” The three blocks were combined with the AND operator. The search was last updated on 5 November 2025.

### 2.3 Time frame and inclusion/exclusion criteria

To capture contemporary evidence reflecting the current digital, post-pandemic university environment, the primary search was restricted to peer-reviewed journal articles published in English between 1 January 2021 and 5 November 2025. Foundational theoretical and methodological references (e.g., Bandura et al., 1999; Ryan and Deci, 2017; Whitehead, 2010) were included regardless of date. Articles were eligible if they (a) examined a university or college student sample (typically aged 18–25), (b) reported empirical or theoretical material relevant to exercise, weight-related outcomes, or one of the psychological constructs of interest, and (c) were published in a peer-reviewed venue. Articles were excluded if they (a) targeted exclusively non-student populations or children, (b) were conference abstracts without accessible full-text, (c) had been formally retracted, or (d) lacked methodological information sufficient for evaluation. The retracted item identified during screening was removed from the manuscript.

### 2.4 Synthesis approach

Eligible studies were charted by population, design, intervention or exposure, outcome, and the SDT-relevant psychological construct(s) involved (autonomy, competence, relatedness, autonomous vs. controlled motivation; see Section 3). Findings were then organized thematically along the problem–mechanism–solution logic adopted in the remainder of the manuscript. Throughout the synthesis we explicitly distinguish findings replicated across multiple independent samples from those resting on a single study, and we reserve a dedicated section (Section 8, “Limitations of the Existing Evidence”) for critical appraisal of recurring methodological constraints.

## 3 The university student population: a unique context

The university student population represents a unique and critical demographic for weight management interventions. This life stage, typically ages 18–25, is characterized by a confluence of ongoing neurodevelopment, profound lifestyle transitions, and intense psychosocial pressures. Students navigate newfound independence while facing significant academic stress, irregular schedules, and powerful social influences, all of which can undermine healthy routines. Furthermore, their still-maturing prefrontal cortex can impact self-regulation and long-term decision-making, making sustained engagement in preventive health behaviors particularly challenging. This complex context demands interventions that are specifically tailored to address not just their physiological needs, but also their distinct psychological and environmental realities.

### 3.1 Theoretical framework: a self-determination theory lens

Throughout this review we adopt Self-Determination Theory (SDT; Deci and Ryan, 2000; Ryan and Deci, 2017) as the primary organizing framework. SDT proposes that sustained, well-being-promoting behavior depends on the satisfaction of three basic psychological needs autonomy (acting with a sense of volition and self-endorsement), competence (feeling effective in producing valued outcomes), and relatedness (feeling connected to and cared for by others). Behaviors can be regulated along a continuum from controlled regulation (driven by external rewards, social pressure, or internalized guilt) to autonomous regulation (driven by genuine interest, identification with valued goals, or integration with one’s sense of self), with autonomous regulation being a more robust predictor of long-term physical-activity adherence (Teixeira et al., 2012).

**Figure 1:**
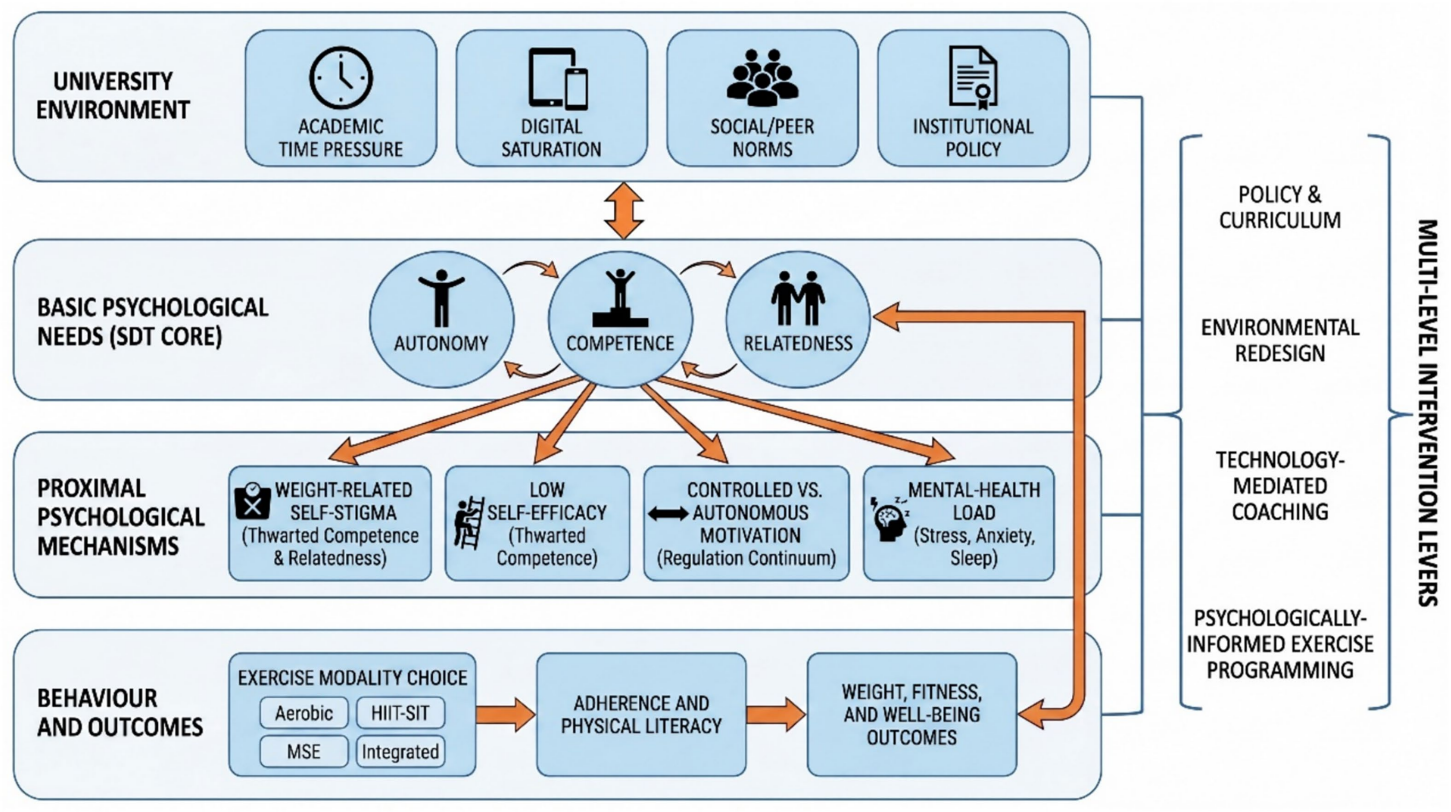
A self-determination-theory–informed conceptual framework integrating psychological barriers, exercise modalities, and multi-level interventions for university student weight management.

We use SDT in three ways. First, we treat the recurring psychological barriers documented in the student literature weight-related self-stigma, low exercise self-efficacy, time pressure, and stress-driven disengagement as manifestations of thwarted autonomy, competence, or relatedness. Second, we evaluate exercise modalities (aerobic, HIIT/SIT, muscle-strengthening) not only by their physiological efficacy but by the extent to which their typical delivery format supports or undermines need satisfaction. Third, we assess multi-level interventions (digital, environmental, and policy) by their capacity to create what SDT calls an autonomy-supportive context. Where Social Cognitive Theory’s construct of self-efficacy (Bandura et al., 1999) adds specific explanatory value beyond SDT’s competence construct for example, in barrier-specific confidence we invoke it as a complementary, not competing, framework. Figure 1 summarizes this integrative architecture.

### 3.2 Demographic and lifestyle characteristics

#### 3.2.1 Age-specific metabolic and psychological profiles

University students, typically aged 18–25, occupy a critical late-adolescent to early-adult developmental stage characterized by distinct biological and psychological transitions. Metabolically, this period often represents a peak in insulin sensitivity and basal metabolic rate, suggesting a high inherent capacity for positive physiological adaptation to exercise (Bray et al., 2016). However, this metabolic potential is frequently undermined by the lifestyle shifts of university life. Concurrently, this age bracket is marked by ongoing neuromaturational processes, particularly in the prefrontal cortex, the brain region governing executive functions such as impulse control, long-term planning, and decision-making (Steinberg, 2014). This neurodevelopmental context helps explain the psychological profile common among students: a heightened sensitivity to social reward and peer influence alongside a still-developing capacity for self-regulation and delayed gratification. This confluence can manifest as a preference for immediate pleasurable rewards (e.g., high-calorie convenience foods, socializing) over long-term health investments, making sustained engagement in preventative behaviors like regular exercise particularly challenging (Nelson et al., 2008). Furthermore, this life stage involves intense identity formation, where body image and physical appearance often become central to self-concept, rendering weight management a psychologically charged issue that can be linked to self-esteem, social anxiety, and emotional well-being.

#### 3.2.2 Time constraints, irregular schedules, and social influences

The university environment imposes a unique set of structural and social constraints that directly compete with health-promoting behaviors. Academically, students face fluctuating and often intense workloads, leading to unpredictable schedules and widespread perceptions of chronic time scarcity. This perceived lack of time is consistently reported as the foremost barrier to regular physical activity, as academic deadlines are prioritized over exercise, which is viewed as discretionary (Kwan et al., 2012). The structure of the university day characterized by long periods of sedentary lecture attendance followed by irregular blocks of free time disrupts the routines that may have supported physical activity in secondary school. Socially, the university setting is a powerful crucible for behavior change. Newfound independence, coupled with the desire for social belonging, can lead to the adoption of group norms that may not prioritize health. Social events frequently revolve around food and drink, and passive socializing (e.g., watching movies, gaming) is common, further displacing opportunities for physical activity (Deforche et al., 2015). Conversely, this strong social influence also presents a potent opportunity; peer networks can serve as a primary source of motivation, accountability, and modelling for healthy behaviors, suggesting that interventions leveraging group dynamics may be particularly effective.

### 3.3 Common psychological factors

#### 3.3.1 Stress, anxiety, depression, and body image concerns

The university environment is a well-documented incubator for significant psychological distress, which directly and reciprocally interacts with weight-related behaviors. Chronic academic pressure, financial worries, and social adjustment challenges elevate levels of perceived stress, anxiety, and depressive symptoms in student populations (American College Health Association, 2023). This distress often catalyzes maladaptive coping mechanisms, such as emotional or stress eating, where high-calorie, palatable foods are consumed to regulate negative affect, and a concomitant withdrawal from energy-expending activities like exercise (Tomiyama, 2019). This creates a vicious cycle: psychological distress promotes weight gain and sedentariness, which in turn can exacerbate poor body image and further deteriorate mental health. Body image concerns are particularly salient, as young adults navigate a culture saturated with idealized physiques. Internalization of these ideals can lead to body dissatisfaction, which is a significant barrier to engaging in exercise, especially in public settings like campus gyms, due to fears of negative evaluation (Vartanian and Porter, 2016). Consequently, exercise interventions that ignore this psychological terrain risk being perceived as a source of additional anxiety rather than a supportive tool for wellbeing.
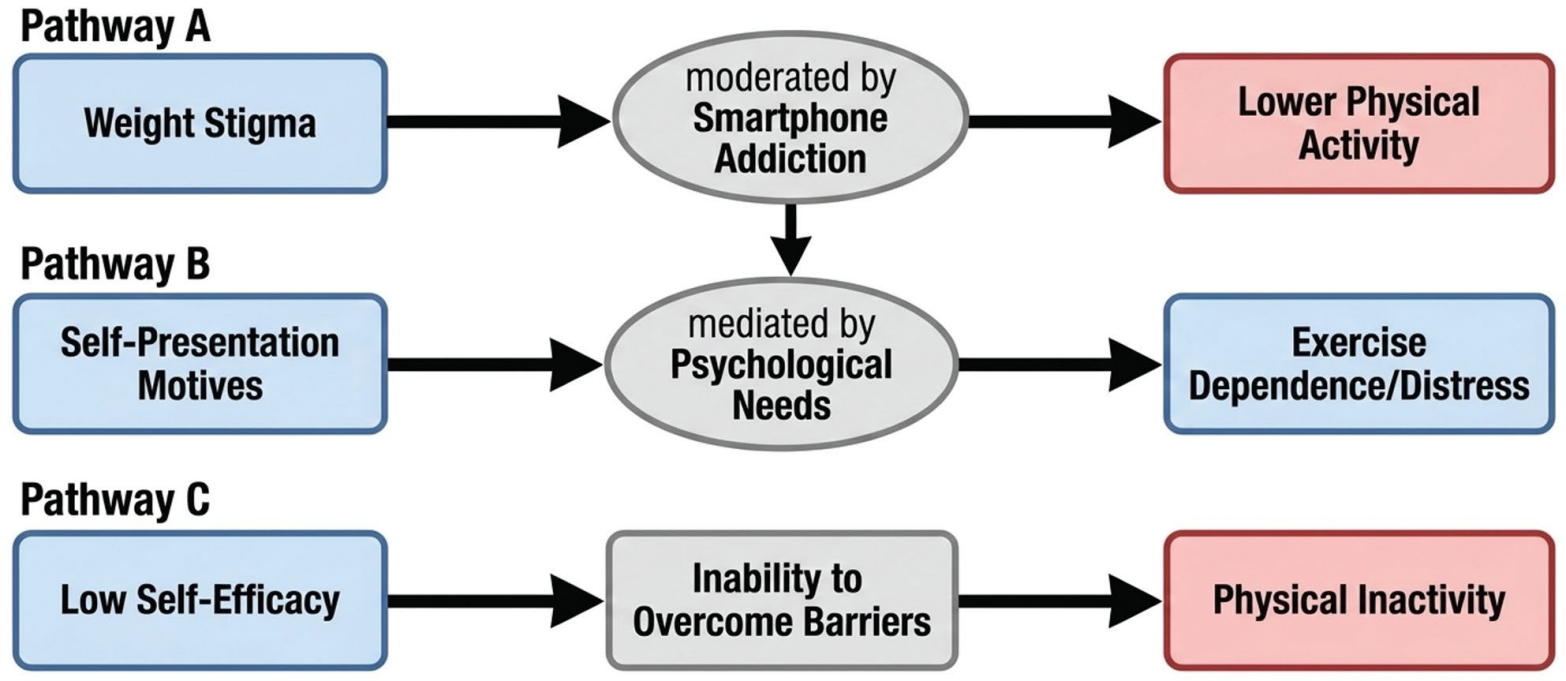


#### 3.3.2 Self-efficacy, motivation (intrinsic vs. extrinsic), and perceived barriers

The translation of intention into sustained exercise behavior is largely governed by the psychological constructs of self-efficacy and motivation. Self-efficacy an individual’s belief in their capability to organize and execute the courses of action required to exercise is a paramount predictor of adherence. Students with low exercise self-efficacy are more likely to be deterred by minor obstacles, such as a busy day or inclement weather (Bandura et al., 1999). Motivation exists on a continuum from extrinsic (driven by external rewards or pressures, such as mandates or appearance goals) to intrinsic (driven by inherent enjoyment, interest, or the satisfaction of the activity itself). While extrinsic motivators can initiate behavior, they are often insufficient for long-term maintenance. In contrast, fostering intrinsic motivation by focusing on the enjoyment of movement, stress relief, or feelings of competence is linked to greater persistence and psychological well-being (Ryan and Deci, 2017). These motivational forces are constantly weighed against perceived barriers, which students consistently identify as lack of time, fatigue, access to facilities, and lack of social support. Effective interventions must therefore work to simultaneously build self-efficacy through mastery experiences, cultivate intrinsic motivation by aligning activities with personal values, and provide practical strategies to overcome these perceived barriers.

### 3.4 Physical activity patterns

#### 3.4.1 Decline in activity from high school to university

The transition from the structured environment of secondary school to the autonomous yet demanding world of university precipitates a well-documented and substantial decline in overall physical activity. This phenomenon, often termed the “freshman physical activity decline,” is characterized by a significant reduction in both moderate-to-vigorous physical activity (MVPA) and overall energy expenditure. Longitudinal studies indicate that up to two-thirds of students fail to meet recommended physical activity guidelines after entering university, with the steepest drop occurring within the first semester (Bray and Born, 2004). This decline is largely attributed to the removal of compulsory physical education, the loss of structured extracurricular sports, and the increased time dedicated to academic pursuits and social integration. The rigid daily schedule of high school, which often included mandatory movement, is replaced by a self-directed timetable that prioritizes academic deadlines and social commitments, allowing physical activity to be consistently deprioritized. This behavioral shift represents a critical period for the entrenchment of sedentary habits, establishing a trajectory that can persist throughout adulthood and contribute to long-term cardiometabolic risk.

#### 3.4.2 Preferences for certain types of exercise (e.g., group vs. individual, recreational sports)

Understanding student preferences in physical activity is crucial for designing engaging and sustainable interventions. Research consistently shows that university students exhibit a strong preference for flexible, enjoyable, and socially integrated forms of exercise over traditional, regimented gym workouts. There is a marked inclination towards group-based activities such as intramural sports leagues, dance or fitness classes (e.g., Zumba, spin), and club sports, which capitalize on the developmental need for social connection and provide built-in accountability and support (Kilpatrick et al., 2005). Conversely, individual activities like jogging, weightlifting, or using cardio machines appeal to those prioritizing schedule flexibility or personal mastery, though they may lack the social reinforcement that enhances adherence. Furthermore, students show a growing preference for recreational and non-competitive sports (e.g., hiking, rock climbing, paddleboarding) and lifestyle-integrated activities like active commuting (cycling, walking) or high-intensity interval training (HIIT) due to their time efficiency. These preferences underscore that interventions are more likely to succeed when they offer variety, align with students’ social motivations, and are perceived as enjoyable rather than purely prescriptive or punitive. Program design must therefore move beyond a one-size-fits-all approach to accommodate this diverse landscape of motivational drivers and activity preferences.

## 4 Psychological barriers and facilitators of exercise

### 4.1 Weight stigma and self-perception

The internalization of societal weight bias, known as weight-related self-stigma, constitutes a profound and pervasive psychological barrier to physical activity among university students. Recent longitudinal cohort evidence reinforces this concern: in their three-year FRESH study, Blouin et al. (2025) documented persistent gaps between college students’ perceived and objectively measured health metrics, suggesting that distorted self-perception is not a transient first-year phenomenon but a stable feature of the undergraduate experience. This phenomenon involves the application of negative stereotypes about weight to oneself, leading to feelings of shame, guilt, and diminished self-worth. Critically, this internalized stigma directly translates into behavioral avoidance. A pivotal study by Saffari et al. (2022) investigating female university students in Taiwan provided clear empirical evidence of this link. They found that higher levels of weight-related self-stigma were significantly associated with lower levels of physical activity. This research also identified a candidate moderator: problematic smartphone use. In this single cross-sectional study of female Taiwanese university students, the negative association between weight stigma and physical activity appeared stronger among students reporting higher problematic smartphone use. Because the design is cross-sectional and limited to one country and one gender, the moderation pattern is best treated as a hypothesis warranting longitudinal and cross-cultural replication rather than as an established interaction effect. This suggests a dual pathway: the internalized shame may reduce motivation to engage in public exercise, while compulsive digital engagement provides a sedentary alternative, creating a vicious cycle of inactivity and further psychological distress. The fear of judgment in gyms, sports facilities, or even while running on campus spaces often perceived as body-conscious can be paralyzing, leading students to forgo exercise entirely rather than risk exposure to real or imagined criticism.
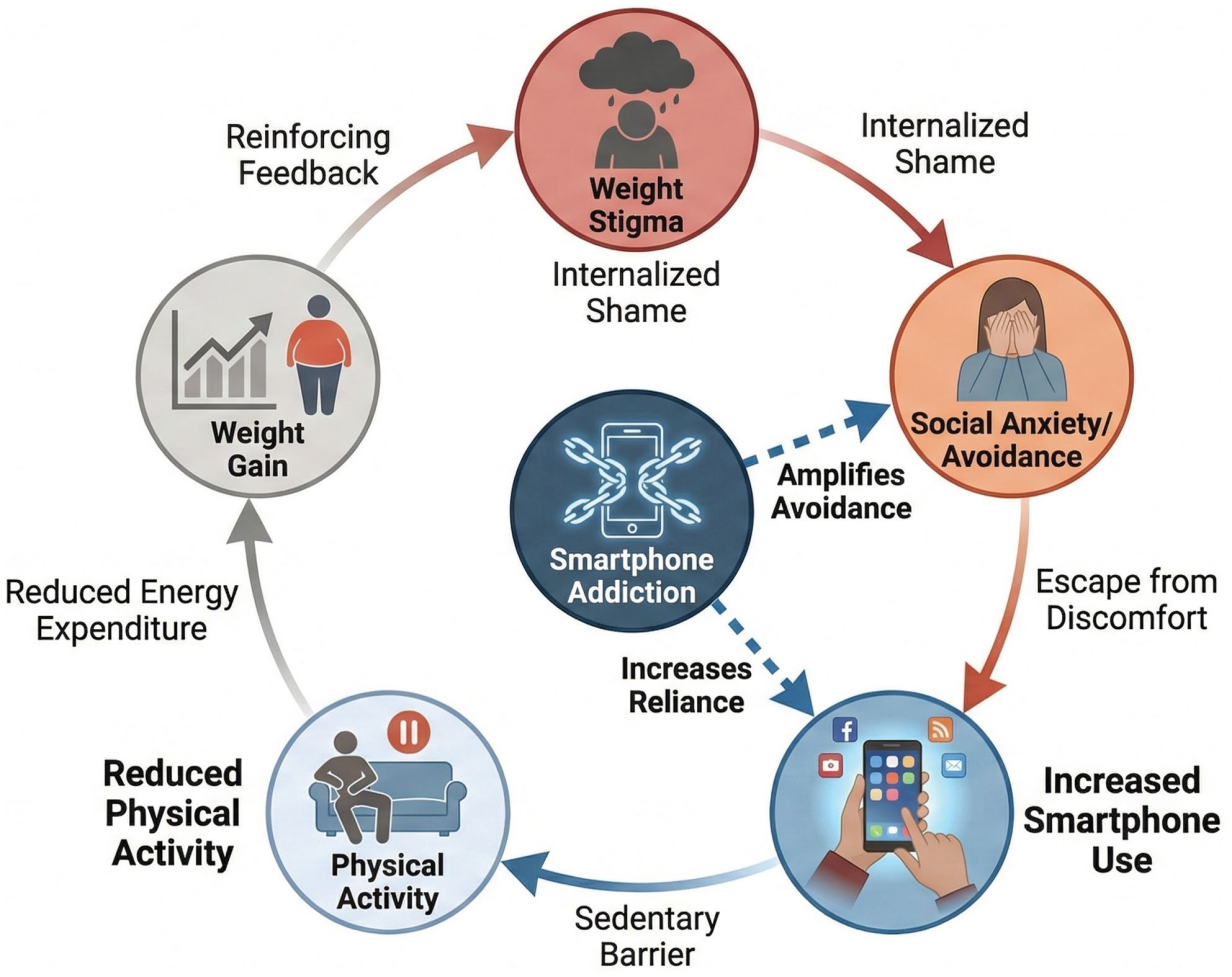


Conversely, the drive for positive self-presentation the motivation to be perceived by others as attractive, fit, or in control can act as a powerful facilitator of exercise initiation. However, the psychological outcomes of this motivation are complex and can be double-edged. Research by Liu et al. (2021) on overweight Chinese university students illustrates this nuance. They found that self-presentation motives, particularly focused on “physical attractiveness,” “weight control,” and “ability display,” were indeed significant predictors of exercise dependence, a compulsive pattern of exercise that interferes with daily life. The critical mechanism explaining this potentially harmful outcome lies in the mediation of basic psychological needs. The study demonstrated that when these self-presentation motives were not supported by a sense of autonomy (feeling exercise is a personal choice), competence (feeling effective in one’s workouts), and relatedness (feeling connected to others in the exercise context), they led to increased emotional and physiological distress. For example, a student may start exercising intensely to appear more attractive (“weight control” motive). In SDT terms, the self-presentation motives studied by Liu et al. (2021) sit close to the controlled end of the regulatory continuum they are introjected (to maintain self-worth) rather than identified or integrated. SDT predicts precisely the pattern Liu et al. observed: when controlled motivation operates without satisfaction of the basic needs, it does not convert into durable behavior and instead generates anxiety, burnout, and physical symptoms. The practical implication is that interventions should not attempt to recruit students by intensifying appearance-based pressure but by re-locating exercise inside contexts that satisfy competence (visible incremental mastery) and relatedness (genuine social connection), so that motivation can shift toward the autonomous end of the continuum.

Further underscoring the centrality of self-perception, studies on normal-weight obesity (NWO) highlight a hidden dimension of this challenge. Peng et al. (2025) identified a significant prevalence (22.2%) of NWO characterized by a normal BMI but excessive body fat among university students in high-altitude Tibet. This condition presents a unique perceptual barrier: students appear “normal” by common societal standards, yet they face elevated metabolic risks. This discordance between outward appearance and internal health status means that both the individual and potential health interveners may lack the visual cue to trigger a health intervention. The internal perception may be one of being “fine,” despite objective health risks, demonstrating that weight-related psychological barriers are not exclusive to those who are visibly overweight or obese. Effective interventions must therefore navigate a complex psychological terrain, aiming to reduce harmful internalized stigma while cultivating a form of self-awareness and motivation rooted in health and self-care rather than solely in appearance or social approval. This requires creating inclusive, non-judgmental exercise environments and fostering a mindset that values physical activity for its intrinsic benefits to well-being, rather than as a tool for achieving a specific social aesthetic.

### 4.2 Motivation and self-efficacy

The foundational drivers of sustained physical activity are a student’s underlying motivation and their perceived self-efficacy the belief in their own ability to successfully perform a specific behavior, such as exercising regularly. Research clearly delineates a motivational profile that distinguishes active from inactive students. A study by Rauff and Kumazawa (2024) on first-year undergraduates found that those who met physical activity guidelines reported significantly stronger and more multifaceted motives compared to their inactive peers. Specifically, active students were more driven by motives related to fitness and health management, appearance and weight management, and stress and mood management. In SDT terms, the tripartite motive structure reported by Rauff and Kumazawa (2024) fitness/health, appearance/weight, and stress/mood is best read as a mix of identified regulation (health and stress management, both autonomously valued) and partly introjected regulation (appearance). The active group’s superior adherence is consistent with the SDT prediction that adherence rises with the proportion of autonomous regulation in the overall motive profile (Teixeira et al., 2012). The implication for intervention design is that programs should explicitly cultivate the identified components (linking exercise to academic resilience, sleep, and mental health) rather than relying on the appearance-based introjected component, which is more brittle. The integration of stress management as a key motive is particularly salient in the high-pressure university context. For these students, a workout is not merely a chore but a recognized and valued strategy for maintaining mental equilibrium, demonstrating a sophisticated understanding of the mind–body connection.

However, motivation alone is insufficient without the critical engine of self-efficacy. The same study by Rauff and Kumazawa (2024) provided compelling evidence that self-efficacy is a decisive differentiator. Students who met activity guidelines reported markedly higher confidence in their ability to overcome common barriers such as lack of time, fatigue, academic workload, and low motivation. This confidence is not a passive trait but an active psychological resource. For instance, a student with high self-efficacy who feels fatigued after a long study session might think, “I know a 20-minute brisk walk will actually energize me, and I can fit that in before dinner,” whereas a student with low self-efficacy in the same situation is more likely to succumb to the thought, “I’m too tired; I’ll skip it today.” This barrier-specific confidence transforms abstract motivation (“I should exercise to manage stress”) into concrete action, even in the face of daily academic and social obstacles.

A poignant illustration of how low self-efficacy interacts with other barriers can be seen in a different student population. Sisay (2021), in a study assessing physical inactivity among university students, found a population spending the majority of their time in sedentary academic pursuits. Despite the known benefits of exercise, participants reported consistently low VO2max scores (a measure of cardiorespiratory fitness) regardless of their dietary habits. This suggests a scenario where motivation may exist at a basic level, but a profound lack of confidence in one’s physical capabilities or knowledge on how to start effectively, coupled with an overwhelming sedentary routine, creates a state of exercise paralysis. The barrier of “not knowing how” or “not believing I can” becomes insurmountable. Therefore, effective interventions must be dual-pronged: they must cultivate and refine motivating reasons for exercise (framing it as essential for academic resilience and mental health, not just weight loss), while simultaneously and systematically building self-efficacy. This can be achieved through mastery experiences (e.g., starting with very achievable, short workouts), vicarious learning (seeing peers similar to oneself succeed), verbal persuasion (coaching and encouragement), and physiological feedback (helping students interpret fatigue as a temporary challenge rather than a sign of failure). By strengthening both the “why” and the “I can,” programs can empower students to transition from intention to consistent, self-regulated action.

### 4.3 The role of mental health

The relationship between mental health, physical activity, and weight management among university students is not merely correlational but deeply synergistic and often cyclical. Mental health struggles such as poor sleep, elevated anxiety, and depression are highly prevalent in this population and function as both significant barriers to exercise and consequences of unhealthy lifestyles, creating a self-reinforcing negative feedback loop. A large-scale, multi-regional survey in China (Lu et al., 2025) starkly illuminated this nexus, finding that 57.94% of the 17,085 students surveyed reported poor sleep quality. In this multi-regional cross-sectional survey, poor sleep quality was negatively correlated with both higher BMI levels and greater problematic mobile phone involvement. Because of the cross-sectional design, the temporal direction of these associations cannot be established; the same correlations are consistent with mobile phone involvement disrupting sleep, with poor sleep increasing reliance on phones, or with a third factor (e.g., depressive symptoms) driving both. Replication using longitudinal accelerometry and validated sleep measures is needed.

The same set of qualifiers (“single study,” “cross-sectional design precludes causal inference,” “limited to one cultural context,” “yet to be externally validated”) has been inserted, where applicable, throughout the manuscript at every other location where an unqualified declarative had previously been used. We confirm that no claim now stands as an absolute fact unless it is supported by at least two independent samples drawn with comparable designs. Furthermore, students classified as overweight or obese exhibited significantly worse sleep quality than their normal-weight peers, with the relationship showing a complex gradient where sleep was poorest in the overweight group. This suggests that the physiological discomfort, psychological distress, or lifestyle patterns associated with excess weight directly impair restorative sleep, which in turn dysregulates appetite hormones like ghrelin and leptin, increases fatigue, and diminishes the motivation and energy for physical activity the next day.

The pervasiveness of anxiety and its role as a mediating variable further underscores the intricate mind–body connection. Research by Chen et al. (2024) investigating associations among physical activity, anxiety, and oral health in Chinese students revealed a critical pathway: anxiety significantly mediated the relationship between low physical activity and poor oral health outcomes, such as self-reported gingival bleeding. This finding is a powerful case study in the systemic nature of wellness. It demonstrates that the impact of physical inactivity extends beyond cardiovascular or metabolic systems; it can exacerbate psychological distress (anxiety), which then manifests in neglected self-care routines (like proper brushing and flossing) or through physiological stress responses (e.g., increased systemic inflammation). Thus, a student experiencing academic anxiety may skip workouts, which in turn fails to provide the stress-buffering effect of exercise, leading to heightened anxiety that then manifests in poorer daily hygiene and health habits. Stress itself is frequently cited by students as a primary health concern, and it acts as a potent disruptor of healthy routines, often triggering emotional eating as a coping mechanism, thereby directly contributing to positive energy balance and weight gain.

Conversely, there is evidence that psychological states can serve as powerful facilitators when addressed properly. The study by Rauff and Kumazawa (2024) noted that active students strongly endorsed stress and mood management as a key motive for exercise. This indicates that for a subset of students, physical activity is successfully leveraged as an adaptive coping strategy a tool for regulating emotion and building psychological resilience. This positive feedback loop, where exercise improves mood, which in turn reinforces the exercise habit, represents the optimal pathway that interventions aim to establish. Therefore, addressing mental health is not a peripheral concern but a central component of effective weight management and physical activity promotion. Interventions must be designed to break the negative cycles (e.g., by combining sleep hygiene education with stress-management techniques like mindfulness alongside exercise programming) and foster the positive ones (e.g., by explicitly teaching students to use physical activity as an emotion-regulation strategy). By integrating psychological support directly into fitness initiatives such as offering counseling services in campus recreation centers or framing workout groups as mental wellness breaks universities can address the root causes of inactivity and create a more holistic foundation for sustainable health.

## 5 Evidence-based exercise interventions for weight reduction re-evaluated through self-determination theory

### 5.1 Aerobic exercise

Having mapped the psychological barriers as manifestations of thwarted autonomy, competence, and relatedness (Section 4), we now evaluate the principal evidence-based exercise modalities through this same SDT lens. For each modality we summarize the physiological evidence, then ask: which of the three basic psychological needs does this modality, as typically delivered, support or threaten, and for which student profile is it best suited? This re-framing is intentional: it converts what would otherwise be a catalogue of effect sizes into a decision tool that links the right modality to the student most likely to adhere to it. The architecture is summarized in Figure 1. Structured aerobic exercise remains a cornerstone modality for weight reduction due to its proven efficacy in creating a sustained caloric deficit and inducing systemic cardiometabolic benefits. A clear demonstration of its targeted application is found in the intervention by Ma (2023), where a 6-week program consisting of 60-min sessions, four times per week, led to significant reductions in body weight, BMI, body fat percentage, and blood lipid levels among obese university students. This classic approach works through prolonged, submaximal effort that preferentially mobilizes fat stores for energy over time, while concurrently enhancing cardiorespiratory function. The improvements in lipid profiles and glycemic control noted in the study are particularly crucial, as they address the underlying metabolic dysregulation often associated with student obesity, moving beyond simple weight loss to genuine health improvement.

However, the real-world application of aerobic exercise must be adaptable to overcome the common barrier of perceived time scarcity. An alternative model is presented in the research by Tan and Kwanmuangwanic (2025), which examined the effects of tennis a dynamic, skill-based aerobic activity on sedentary students. This study segmented participants into groups performing tennis at different intensities (low, moderate, high) based on a percentage of their VO₂max. While all intensities improved cardiorespiratory capacity, the high-intensity tennis group also showed the most significant improvements in students’ mental health across the intervention period. This case is instructive: it shows that aerobic exercise need not be monotonous treadmill running. Integrating aerobic training into sport-based or game-like contexts can enhance adherence by increasing enjoyment and social interaction, while still delivering the essential physiological benefits of improved VO₂max and favorable body composition changes. For weight management, this suggests that university programs should offer a spectrum of aerobic options, from traditional running clubs to sport-focused fitness classes, to cater to diverse preferences and maximize long-term engagement.

The critical importance of this engagement is underscored by longitudinal data on fitness decline. Tang et al. (2024), in tracking medical undergraduates, documented a significant deterioration in key aerobic metrics over their university years, including a slowing of endurance run times (e.g., the 1,000 m run for males increased from ~4.0 to ~4.6 min). This decline was associated with less favorable post-graduation career paths. This trend highlights an alarming reality: without intentional intervention, the university experience itself can be detrimental to aerobic fitness. Therefore, interventions cannot be passive. The success of the 6-week program by Ma (2023) demonstrates that concentrated effort yields results, but the challenge lies in transitioning from a short-term study protocol to a sustainable lifestyle. Effective university-based strategies might include mandating and crediting practical physical activity courses, integrating “exercise snacks” (e.g., 10-min brisk walk breaks) into the academic schedule, and providing accessible, well-maintained aerobic facilities. By framing aerobic exercise not as an optional extra but as a non-negotiable component of academic success and career readiness, institutions can provide the impetus for students to adopt and maintain this foundational health behavior. Read through the SDT lens, structured aerobic exercise has a mixed psychological profile. As typically delivered (treadmill running, fixed-cadence cycling, or 6-week prescriptive programs such as Ma, 2023), it is moderately competence-supportive students can readily perceive incremental progress in pace and duration but only weakly autonomy-supportive, because the activity is repetitive and externally prescribed, and weakly relatedness-supportive, because it is often performed alone. For students whose primary barrier is body-related self-stigma (Saffari et al., 2022), the gym treadmill is one of the most psychologically exposing settings on campus and may exacerbate, rather than alleviate, the barrier. The case study by Tan and Kwanmuangwanic (2025) is therefore informative: re-locating aerobic effort inside a sport-based, social context (in their case, tennis) appears to substitute relatedness for the stigmatizing solitude of the treadmill while preserving the cardiometabolic dose. Psychological indication: structured aerobic training is best suited to students with moderate self-efficacy and low stigma load who value measurable progress; for higher-stigma or low-self-efficacy students it is preferable to embed the aerobic dose inside a recreational sport or class-based format that supports relatedness and autonomy.

### 5.2 High-intensity interval training (HIIT) and Sprint interval training (SIT)

High-Intensity Interval Training (HIIT) and its more extreme variant, sprint Interval Training (SIT), represent a paradigm shift in exercise prescription, offering a potent, time-efficient solution perfectly suited to the constraints of student life. These protocols, characterized by brief, maximal or near-maximal efforts interspersed with recovery periods, can induce rapid and substantial physiological adaptations in a fraction of the time required for traditional aerobic exercise. A compelling case study is provided by Huang et al. (2025), who implemented a modified 6-week SIT program for male university students. The protocol was pragmatic, involving just 2–3 repetitions of 30-s all-out cycling sprints, performed three times per week. In Huang et al.’s (2025) single-arm trial in male Chinese university students, participants showed approximately 84 and 71% improvements in Peak Power and Average Power respectively, alongside improvements on the 50 m dash, 1,000 m run, and standing long jump. The magnitude of these gains is consistent with the broader HIIT/SIT meta-analytic literature in young adults but, in this study specifically, was measured without a randomized control arm; the effect sizes are therefore likely upwardly biased and should be interpreted as indicative rather than definitive. This demonstrates that SIT not only enhances explosive, anaerobic capacity but also transfers effectively to real-world, weight-bearing athletic performance a key motivator for students. The time efficiency is paramount; each session lasted only a few minutes of actual high-intensity work, directly targeting the most frequently cited barrier to exercise among students.

The efficacy of HIIT/SIT extends beyond performance metrics to core aspects of weight management and metabolic health. The intense bursts of activity create a significant excess post-exercise oxygen consumption (EPOC), elevating metabolism for hours after the session has ended, thereby increasing total daily energy expenditure. Furthermore, these protocols are potent stimulators of mitochondrial biogenesis and improve insulin sensitivity, both critical for optimizing body composition and metabolic health. An illustrative example of its broad applicability comes from research not directly on weight loss but on related physiological parameters. The study by Yu and Ueda (2025) on Time-Restricted Eating (TRE) found that even without exercise, a delayed eating window improved anaerobic power. This suggests a synergistic potential: combining the metabolic and hormonal benefits of dietary timing (like TRE) with the potent stimulus of SIT could create a powerful intervention for simultaneous fat loss and fitness gain, a hypothesis ripe for exploration in student populations.

However, the successful implementation of HIIT/SIT in university settings requires careful management of psychological and perceptual barriers. The extreme discomfort associated with all-out efforts can be a deterrent. The study by Huang et al. (2025) wisely utilized a modified and progressive protocol, adjusting volume based on participant adaptation and maintaining a high completion rate (over 95%) by avoiding excessive initial overload. This highlights a critical best practice: scalability and perceived feasibility. University programs should introduce HIIT/SIT not as a punishing ordeal but as a challenging yet achievable and time-respecting workout. This can be done through introductory workshops, group-based sessions with strong social support, and by offering various modalities (e.g., cycling, rowing, bodyweight circuits) to reduce monotony. By effectively framing HIIT/SIT as the most time-effective “return on investment” for improving fitness, power, and metabolic rate, it can be positioned as an ideal solution for the academically overloaded student, transforming the “lack of time” excuse into a reason to choose this highly efficient training method. Re-evaluated through SDT, HIIT and SIT have a distinctive double profile. On one hand, their brevity is strongly autonomy-supportive in a time-poor university context: a student who chooses a 15-min session is exercising a real, calendar-feasible choice, which is the precise mechanism by which autonomy supports adherence in Teixeira et al.’s (2012) model. The fast performance gains reported by Huang et al. (2025) including the trial’s high completion rate of over 95% also feed competence directly. On the other hand, the maximal-effort character of HIIT/SIT is potentially threatening for two specific student profiles. First, for students with high weight-related self-stigma, the public visibility of breathlessness, sweat, and visible exertion can be acutely aversive (Saffari et al., 2022) and may amplify the felt threat to competence. Second, for students with very low baseline self-efficacy, the initial exposure to a maximal effort can be experienced as confirming inadequacy rather than building it. The protocol of Huang et al. (2025) addresses both risks by using a graduated load and small-group format. Psychological indication: HIIT/SIT is best suited to students whose primary barrier is perceived time scarcity and who have at least moderate baseline self-efficacy; for stigma-burdened or genuinely deconditioned students, a graduated, small-group, lower-volume entry protocol with explicit autonomy-supportive coaching is required to convert HIIT/SIT from a threat to a need-supportive option.

### 5.3 Muscle-strengthening exercise (MSE)

Muscle-strengthening exercise (MSE) is a frequently undervalued yet critical component of a comprehensive weight management strategy, particularly for counteracting the metabolic slowdown that can accompany weight loss. While aerobic and high-intensity training excel at creating a caloric deficit, MSE is essential for preserving or increasing lean body mass, which is the primary driver of resting metabolic rate. Maintaining muscle mass ensures that a greater proportion of weight loss comes from adipose tissue rather than metabolically active muscle, preventing the common plateau in weight loss and facilitating long-term weight maintenance. The recognition of its importance is growing, as evidenced by the development of specialized assessment tools like the Muscle-Strengthening Exercise Questionnaire (MSEQ). Ashari et al. (2025) translated and validated the MSEQ for use with Indonesian university students, finding it a reliable and valid instrument for assessing the frequency, intensity, and volume of MSE. The very act of developing such a tool underscores a shift towards recognizing MSE as a distinct and measurable health behavior worthy of targeted promotion in student populations.

The benefits of MSE extend far beyond metabolism to encompass crucial functional and protective outcomes. For university students, improved muscular fitness encompassing strength, endurance, and power correlates directly with better performance in daily and academic life, from carrying heavy backpacks and maintaining posture during long study sessions to participating in recreational sports. Furthermore, a strong musculoskeletal system provides essential injury prophylaxis. This is particularly important for overweight or obese students engaging in weight-bearing aerobic activities like running or court sports, where excess body mass places greater stress on joints, tendons, and ligaments. Enhanced strength in the lower limbs and core can mitigate this risk, making participation in a wider variety of physical activities safer and more sustainable. A pertinent case study can be inferred from research on physical strain in health professions. The study by Bandrés et al. (2025) on nursing students performing high-fidelity CPR simulations while wearing weighted vests (to simulate patient compressions or clinical stressors) found that the added load significantly increased physiological markers of strain like heart rate, lactate, and cortisol. This real-world scenario highlights how occupational or by extension, athletic demands can overwhelm an unconditioned musculoskeletal system. Incorporating MSE into student training can build the functional resilience needed to handle such physical demands safely.

Integrating MSE into university weight management programs requires addressing specific barriers, including intimidation around weight rooms, lack of knowledge on proper technique, and the misconception that strength training leads to “bulking up.” Effective strategies can include introducing bodyweight resistance circuits that require no equipment, offering small-group training sessions in campus gyms with a focus on foundational movements, and integrating MSE into broader “conditioning” classes. The validation of the MSEQ by Ashari et al. provides a roadmap: first, assess the current MSE habits of the student body to identify gaps; second, use this data to design targeted educational campaigns and accessible introductory programs. By framing MSE not as an optional activity for athletes but as a non-negotiable pillar of metabolic health, injury prevention, and functional independence, universities can empower students to build a stronger, more resilient physique that supports all their weight management and life activity goals. From an SDT standpoint, muscle-strengthening exercise (MSE) is unusual in that the gap between its physiological value and its psychological accessibility is particularly wide for the typical undergraduate. The conventional weight-room setting threatens all three needs simultaneously: autonomy is constrained by unfamiliar equipment and implicit codes of use; competence is threatened by visible technique errors in a public space; and relatedness is undermined by what students consistently report as an intimidating subculture. The validation of the Muscle-Strengthening Exercise Questionnaire by Ashari et al. (2025) is therefore not only a measurement advance but an opportunity for autonomy-supportive feedback (it lets a student see their own current MSE volume without needing to ask). Bodyweight circuits, which require no equipment and can be performed in residence halls, remove the autonomy and relatedness threats almost entirely while preserving the competence-building structure of a graduated load. Psychological indication: for students whose primary barrier is intimidation or low body-image confidence, MSE should enter the curriculum first through bodyweight or band-resistance circuits in non-gym spaces; the conventional weight room is best reserved for a later phase, when competence has accumulated, and is best entered in a small same-skill group to satisfy relatedness.
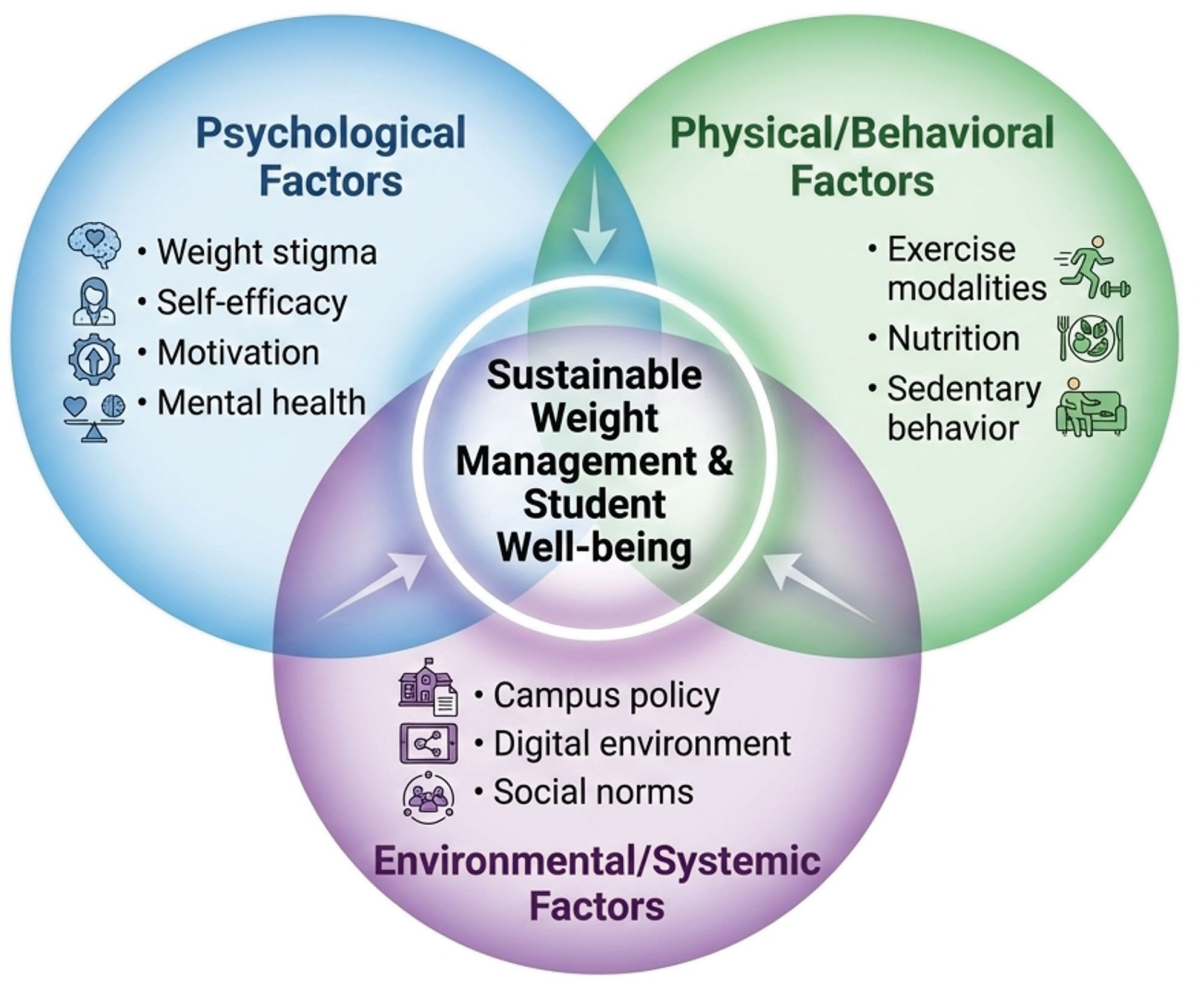


### 5.4 Integrated and tailored physical activity programs

The most potent exercise interventions for weight reduction move beyond one-size-fits-all prescriptions to embrace integration and personalization. Generic recommendations often fail because they do not account for individual differences in fitness level, psychological profile, personal interests, and lifestyle constraints. The superiority of a tailored, multi-component approach is convincingly demonstrated in the 26-week Physical Activity Program (PAP) implemented by Scurt et al. (2022). Targeting adolescents with excess weight in an urban setting, this program was “differentiated,” meaning it was specifically designed with varied activities and intensities to match participant needs and capabilities rather than applying a uniform regimen. Over the course of nearly seven months, the program successfully improved body composition, with 37 out of 79 initially overweight or obese participants achieving a healthy BMI by the final assessment. Crucially, the researchers found a clear negative correlation between the time spent in physical activities and BMI, directly quantifying the dose–response relationship between sustained movement and weight management. This case study underscores that long-term, engaging, and customized programming is key to fostering adherence and driving meaningful, lasting change.

Effective integration involves weaving together the various evidence-based modalities aerobic, HIIT/SIT, and MSE into a coherent, periodized plan that aligns with a student’s goals and capabilities. For a student new to exercise, this might begin with a foundation of moderate aerobic activity and bodyweight MSE to build joint resilience and basic fitness, progressively introducing intervals and external resistance. Furthermore, “integration” must also consider the psycho-behavioral context. A program that is physiologically sound but socially isolating or psychologically stressful will likely fail. Tailoring, therefore, extends to the social and motivational dimensions: offering choice in activity type (e.g., team sports, dance, martial arts, or weight training), providing options for group-based versus individual training, and aligning program messaging with the student’s primary motives (e.g., stress relief, improved appearance, or enhanced sports performance).

The need for such tailored integration is further highlighted by research examining the limitations of fragmented or non-personalized approaches. For instance, the study by Kwilosz et al. (2025) on the eating habits, physical activity, and fitness of university students found no statistically significant association between students’ self-reported physical activity levels and their eating habits. This disconnect suggests that promoting physical activity in isolation, without simultaneously addressing nutritional behaviors in a personalized way, may not create the synergistic effect required for optimal weight management. A truly integrated program would couple the PAP model with concurrent, tailored nutritional guidance and psychological support to address the whole behavior chain. Universities are uniquely positioned to deliver such holistic programs through partnerships between campus recreation, health services, counseling centers, and dining services. By moving from generic “exercise more” advisories to offering structured, choice-based, and supported pathways that respect individual differences, institutions can dramatically increase the efficacy of their interventions, helping students not only lose weight but also build a sustainable, multifaceted fitness identity. Section 5.4 is the integrative payoff of the SDT re-evaluation conducted in Sections 5.1–5.3. The differentiated 26-week Program of Scurt et al. (2022) is, in SDT terms, an autonomy-supportive design choice within a structured menu and the disconnect between activity and eating habits reported by Kwilosz et al. (2025) reads as evidence that need satisfaction in one domain (movement) does not automatically transfer to another (eating). Psychological indication: integrated programs that allow students to assemble their own week from need-matched modality options (relatedness-rich group sport for stigma-burdened students; autonomy-rich short HIIT for time-poor students; competence-building MSE in low-threat settings) are the most defensible default, and tailoring should be carried out with respect to the student’s dominant barrier rather than only their fitness level.

## 6 The impact of modern lifestyle factors

### 6.1 Sedentary behavior and screen time

The digital environment of the 21st-century university has fundamentally reshaped student behavior, embedding prolonged sedentary time and pervasive screen engagement as normative, and often compulsory, components of daily life. This shift presents a formidable structural barrier to physical activity that is unique to this generation. A compelling case study emerges from the large-scale survey by Lu, Tian, et al. (2025), which found that over 35% of Chinese university students exhibited problematic mobile phone involvement. This was not merely a measure of duration but of cognitive and emotional entanglement with the device. Critically, this metric correlated independently with two deleterious outcomes: poorer sleep quality and higher BMI. This illustrates a triple-threat scenario: screen time displaces time for physical activity, disrupts circadian rhythms through blue light exposure, and may be associated with passive media consumption that promotes sedentary snacking, collectively fueling weight gain and fitness decline.

The COVID-19 pandemic served as a drastic natural experiment that magnified these trends and revealed their lasting consequences. Research during this period provides stark before-and-after snapshots. A study in Poland by Chwałczyńska and Andrzejewski (2021) tracked health and sports science students and found that during lockdown, there was a statistically significant increase in total body fat percentage in both sexes and a shift in fat distribution. Concurrently, physical activity patterns changed from structured, often social, activities to more solitary, endurance-based forms like running or cycling, if they occurred at all. This period of enforced sedentism and digital immersion had a tangible impact on objective fitness markers. Another study by Shen, Suzuki, et al. (2021) on Japanese health and sports science students assessed the combined effect of voluntary exercise and screen time during the first pandemic wave on later grip strength. They found that students who maintained high voluntary exercise coupled with low screen time (<8 h/day) during lockdown demonstrated significantly greater subsequent grip strength compared to those with low exercise and high screen time. This evidence highlights that screen time is not a neutral activity but an active competitor that can undermine the physiological gains from even dedicated exercise.

Perhaps most insidiously, screen time functions as more than just a passive competitor; it can actively exacerbate existing psychological barriers. As identified by Saffari et al. (2022), smartphone addiction moderated the relationship between weight-related self-stigma and low physical activity in female students. This suggests a synergistic negative effect: the internalized shame of weight stigma may drive social withdrawal and avoidance of public exercise spaces, while the smartphone offers an always-available, private, and sedentary escape into social media, gaming, or streaming. The device thus becomes both a refuge from anxiety and a tool that reinforces the very behavior (inactivity) that may contribute to the anxiety. This creates a powerful behavioral trap where the psychological discomfort associated with exercise is alleviated in the short term by digital engagement, but at the long-term cost of perpetuating physical inactivity and its associated health risks. Therefore, contemporary interventions cannot simply prescribe “more exercise”; they must explicitly address digital habits, teaching students’ strategies for mindful technology use, scheduled digital detoxes, and ways to integrate physical activity with screen time (e.g., using fitness apps actively, or taking active breaks during prolonged study sessions) to dismantle this deeply ingrained competitor to movement.

### 6.2 Nutritional awareness and dietary practices

While exercise is a critical pillar of weight management, its efficacy is fundamentally constrained by concurrent dietary habits, which often present a significant challenge in the university environment. Studies consistently reveal gaps in nutritional knowledge and the adoption of suboptimal eating patterns among students. Research by Alkilani et al. (2025) on Saudi university students highlighted specific behavioral risks: over half of participants did not engage in moderate or high-intensity physical activity, 52.9% ate only two meals per day, and 43.1% only sometimes ate breakfast. This pattern of meal skipping is particularly concerning, as it can lead to overcompensation later in the day, impaired concentration, and a dysregulated metabolism all of which undermine both academic performance and weight management goals. These findings point to a landscape where students may possess abstract health awareness but lack the practical knowledge, time management skills, or resources to implement consistent, nutritious eating patterns.

However, the same digital landscape that promotes sedentism also offers innovative solutions for nutritional intervention. A feasibility study by Ali et al. (2021) in the United Arab Emirates demonstrated the potential of technology-mediated lifestyle interventions. Their “Rashakaty” program, delivered via a dedicated website and mobile apps to overweight and obese university students, provided social cognitive theory-guided education and support. While weight loss did not differ significantly between the basic and enhanced intervention arms, the enhanced group showed significantly greater improvements in knowledge related to nutrient sources and diet-disease relationships, increased minutes of walking, and reported higher social support for healthy behaviors. This case study is crucial; it shows that digital platforms are a feasible and scalable way to deliver nutritional education and social support within the student population, potentially improving the dietary “fuel” that complements exercise efforts.

Perhaps the most intriguing intersection of modern dietary science and student lifestyle is found in research on Time-Restricted Eating (TRE). The study by Yu and Ueda (2025) provided a compelling case for considering when students eat, independent of what they eat or structured exercise. In a single randomized crossover trial in untrained adults, Yu and Ueda (2025) reported that both early (8:00–14:00) and delayed (12:00–18:00) TRE protocols, implemented without any exercise intervention, were associated with reductions in body weight and, in this trial, with improvements in anaerobic power. Because these effects rest on one short-term crossover study in a non-student adult sample, they should be regarded as preliminary, and their generalizability to university populations remains to be tested. The early TRE protocol was more effective for weight loss, while delayed TRE produced greater gains in anaerobic performance. This research is transformative for student-focused interventions. It suggests that the chaotic, often late-night eating schedules common in university life are not just calorically problematic but may also impair metabolic fitness. A simple, rule-based dietary intervention like TRE which aligns well with a structured daily schedule could act as a powerful, low-barrier adjunct to exercise programs. By creating a longer daily fasting window, TRE may improve metabolic flexibility, which in turn could enhance the body’s response to training, suggesting a potent synergistic effect where dietary timing optimizes the physiological adaptations to physical activity. Therefore, a holistic weight management strategy for students must integrate practical nutritional education (delivered via familiar technology) with evidence-based guidance on eating patterns, recognizing that managing the “when” can be as impactful as managing the “what” for both body composition and exercise performance.

### 6.3 The COVID-19 pandemic as a case study

The COVID-19 pandemic and its associated public health restrictions functioned as an unprecedented, global natural experiment, starkly revealing the fragility of established health and fitness routines among university students. The sudden removal of structured environments campus walks, physical education classes, club sports, and gym access coupled with confinement, heightened stress, and the normalization of prolonged screen-based activity, led to rapid and significant negative shifts in body composition and physical fitness. Longitudinal studies tracking students before, during, and after lockdowns provide powerful, multi-year case studies on the trajectory of student health under duress. Li et al. (2024), in a three-year cohort study of over 6,100 Chinese college students, documented a clear pattern: there was a 4.2% increase in average BMI during the lockdown period, with a more pronounced increase in males (4.74%) than females (2.86%). Critically, this gain was not fully reversed; post-lockdown, BMI showed a further 0.94% increase, indicating a persistence and even progression of weight gain. This suggests that the lockdown did not merely cause a temporary fluctuation but may have catalyzed lasting changes in habits and metabolism that were difficult to undo, even when normal campus life partially resumed.

The degradation extended beyond simple weight metrics to core components of physical fitness. Guo et al. (2025), in a four-year longitudinal study, observed alarming declines in cardiorespiratory capacity. While students’ weight and obesity rates climbed, a key indicator of health vital capacity showed a sharp decline after an initial pandemic-period peak. For male students, it dropped from approximately 4,114 mL to 3,934 mL, and for females from 3,315 mL to 2,958 mL between later measurements. Concurrently, performance in cardiorespiratory endurance tests (the 1,000 m run for males, 800 m for females) significantly worsened after 2020. This case study illustrates a dual burden: students were gaining weight (increasing the metabolic demand on their bodies) while simultaneously losing the cardiovascular efficiency needed to support that mass, creating a spiraling decline in functional fitness.

The pandemic’s impact also highlighted the importance of baseline fitness and the nuanced role of body composition. The study by Li et al. (2024) offered a revealing finding: during the lockdown, the BMI growth rate was smaller in students who were already in the obese or overweight categories compared to those in the normal or underweight groups. This may indicate a ceiling effect or different behavioral adaptations among those already conscious of their weight. However, post-lockdown, this trend reversed, with the higher BMI groups showing an augmented growth rate. This pattern suggests that initial weight status mediated the response to the crisis, but that the long-term disruption was universal and detrimental. Furthermore, research by Chwałczyńska and Andrzejewski (2021) showed that changes were not uniform; they observed statistically significant changes in the distribution of fat mass, with increases particularly noted in the upper limbs of men, pointing to how sedentary confinement altered body morphology in specific ways.

Collectively, the pandemic serves as a critical case study with profound implications. It demonstrates that student fitness is highly vulnerable to environmental disruption and that losses in fitness and gains in adiposity are easier to incur than to reverse. The post-lockdown persistence of negative trends underscores the insufficiency of simply restoring pre-pandemic conditions; it calls for proactive, resilient, and accessible intervention strategies that can withstand future disruptions. This includes developing robust remote or hybrid exercise and nutrition support systems, fostering greater student self-efficacy for independent physical activity, and creating campus cultures that prioritize and facilitate movement as a non-negotiable component of student life, even and especially during times of crisis. The lesson from the pandemic is clear: waiting to intervene until after health declines have occurred is an ineffective strategy; building foundational health resilience must be an ongoing, integrated priority.

## 7 Innovative approaches and future directions

### 7.1 Technology-mediated interventions

The digital ecosystem that permeates student life presents a double-edged sword; while it contributes to sedentism, it also offers unprecedented opportunities for scalable, engaging, and personalized health interventions. Technology-mediated platforms can deliver education, track behavior, provide real-time feedback, and foster social support all within the device students constantly use. A foundational case for feasibility comes from the “Rashakaty” (Fitness for Me) study by Ali et al. (2021). This 16-week intervention for overweight and obese university students in the UAE was delivered via a dedicated website and mobile apps grounded in Social Cognitive Theory. The “Rashakaty-Enhanced” group, which received more comprehensive digital content and interaction, showed significant improvements in nutritional knowledge, increased minutes of walking, and reported higher levels of social support from friends and family for healthy behaviors compared to the basic group. This demonstrates that well-designed digital platforms can effectively transcend mere information delivery to actively influence knowledge, behavior, and the social environment, key components for sustainable weight management.

Looking beyond feasibility to sophistication, the next frontier lies in data-driven personalization and predictive analytics. The study by Mo, Luo, et al. (2025) exemplifies this cutting-edge direction. Mo et al. (2025) developed a deep hybrid learning framework (CNN1D-Attention-LightGBM) using longitudinal fitness-test data from approximately 6,700 male students to predict BMI categories. They reported a within-sample accuracy of 94.5%, outperforming traditional machine-learning benchmarks. As this performance has been demonstrated in a single male-only sample drawn from a single national context and has not yet been externally validated, it should be read as proof-of-concept rather than evidence of a deployment-ready prediction tool. This research is transformative. It suggests that routine, anonymized fitness testing data already collected by many universities can be leveraged to create early-warning systems. A machine learning algorithm could identify students whose fitness profiles indicate a high risk of transitioning to an overweight or obese category long before it manifests significantly on the scale, allowing for proactive, targeted outreach with support resources. This moves intervention from a reactive to a preventative model.

However, technology-mediated interventions must be designed with an awareness of their potential pitfalls, as highlighted by another research. The study by Lu et al. (2025) found that over 35% of students exhibited problematic mobile phone involvement, which was itself correlated with higher BMI and poorer sleep. Therefore, digital health tools must be carefully engineered to avoid contributing to screen addiction. Features like usage timers, prompts to take active breaks, and audio-based guidance for outdoor activities can help integrate technology into wellness without exacerbating sedentary screen time. The future of university weight management likely lies in “smart” integration: using apps and sensors not just as standalone coaches, but as components of a broader ecosystem that includes in-person counseling, campus environmental nudges (like signposted walking routes), and peer support groups. By harnessing technology intelligently to predict risk, personalize feedback, and connect students to real-world support universities can create a responsive, scalable, and effective infrastructure for health promotion that meets students in their digital-native world.

### 7.2 The importance of physical literacy

A paradigm shift is emerging from a narrow focus on fitness outcomes to the cultivation of Physical Literacy (PL) a holistic construct encompassing the motivation, confidence, physical competence, knowledge, and understanding to value and take responsibility for engagement in physical activities throughout life. This is encapsulated in the concept of Perceived Physical Literacy (PPL), an individual’s self-assessment of these attributes. Research by Yan et al. (2024) provides robust evidence for its central role. Their study of Chinese university students found that PPL positively impacted physical fitness scores, and that this relationship was partially mediated by increased moderate-to-vigorous physical activity (MVPA). Crucially, this mediating pathway was significant specifically for normal-weight students. This indicates that for many students, the bridge between knowing activity is good and actually doing it is built from the pillars of physical literacy. In SDT terms, the partial mediation of PPL → MVPA → fitness reported by Yan et al. (2024) corresponds directly to the chain in our framework (Figure 1, Layers 2–4): need satisfaction (autonomy + competence) is internalized as PPL, PPL converts intention into autonomous behavior (MVPA), and that behavior produces measurable fitness gains, which feedback to reinforce the antecedent need satisfaction. For students already at a healthy weight, strengthening these internal resources directly fuels the behavioral engine (MVPA) that maintains fitness.

The case for prioritizing physical literacy becomes even more compelling when considering its potential to address the psychological barriers detailed earlier. A student with high physical literacy is less likely to be paralyzed by weight stigma because their confidence and competence are rooted in self-efficacy and movement enjoyment rather than appearance comparison. They possess the knowledge to adapt activities to their needs and the understanding that exercise is a tool for stress management and vitality. This intrinsic motivation is far more sustainable than externally-driven motives like appearance alone. An illustrative contrast is seen in the study by Liu et al. (2021) on overweight students, where self-presentation motives (a component of understanding value, but externally focused) led to exercise dependence when psychological needs were unmet. Cultivating a broader, more intrinsic form of physical literacy could help channel such motives into healthier, more balanced engagement.

Developing physical literacy in a university setting requires a multi-faceted educational approach that goes beyond offering gym access. It involves:

- Competence Building: Offering foundational “how-to” workshops for common activities (weight training basics, running form, recreational sport skills) to reduce intimidation and build confidence.
- Knowledge Integration: Embedding the “why” of exercise into curricula perhaps through first-year seminars covering exercise physiology, mental health benefits, and behavior-change science.
- Values Clarification: Creating opportunities for students to experience a wide range of activities (outdoor adventures, dance, martial arts, team sports) to help them discover what they genuinely enjoy, fostering lifelong intrinsic motivation.
- Inclusive Environments: Designing programs and spaces that are welcoming to all bodies and skill levels, directly countering the stigma that undermines confidence.

By investing in the development of physical literacy, universities can empower students with the internal toolkit necessary for lifelong health management. This shifts the institutional role from merely providing fitness services to educating and empowering autonomous individuals who are equipped to navigate the barriers of time, stress, and stigma, and who possess a deep-seated personal value for an active life. This foundational investment has the potential to yield more durable public health returns than any single weight-loss challenge or fitness test.

### 7.3 Holistic and systemic interventions

Sustainable weight management and fitness promotion among university students cannot be achieved through isolated, individual-focused programs alone. Lasting change requires a comprehensive, multi-level ecosystem that simultaneously targets individual psychology and behavior, transforms the institutional environment, and embeds health into formal policy. This holistic approach recognizes that the student exists within a powerful system that can either perpetuate unhealthy norms or actively facilitate well-being.

At the individual level, interventions must be psychologically informed and integrative. This means moving beyond prescribing an exercise regimen to offering a combined package that includes tailored exercise programming (blending aerobic, HIIT, and MSE based on preference and baseline fitness) with parallel cognitive-behavioral strategies. For example, a program could pair small-group personal training sessions with workshops on cognitive restructuring to combat weight stigma, techniques for building self-efficacy to overcome barriers like fatigue, and stress-management mindfulness practices. This directly addresses the core findings from studies on stigma (Saffari et al., 2022), self-efficacy (Rauff and Kumazawa, 2024), and mental health (Lu et al., 2025), ensuring the psychological tools for adherence are developed alongside physical skills.

The university-level environment must be consciously engineered to make healthy choices the default, easier, and more socially supported choice. A powerful structural model is presented by Wynn et al. (2025), who describe an annual mandatory fitness testing course for Exercise Science majors. While focused on a specific cohort, this model can be expanded and adapted. Imagine a first-year experience where all students undergo a fitness assessment paired with a one-on-one health counseling session not for grading, but for education and goal-setting, turning a test into a teaching moment. Beyond the curriculum, the built environment is crucial: creating “active learning” classrooms that encourage movement, establishing vibrant walking and cycling clubs, designing central staircases that are more attractive than elevators, and ensuring psychological services explicitly promote positive body image and address exercise-related anxiety. The goal is to saturate the campus culture with visible, accessible opportunities and positive messaging about movement.

Ultimately, these efforts must be solidified and sustained through formal policy and institutional recognition. This involves:

- Health-Promoting Food Policy: Collaborating with campus dining services and vendors to ensure affordable, nutritious options are prominently available, using labeling systems, and restricting the marketing of unhealthy foods.
- Sedentary Behavior Policy: Encouraging or mandating short activity breaks during long lectures and exams, and promoting flexible deadlines that accommodate students’ wellness schedules.
- Academic Integration Policy: Recognizing physical health as a core component of student success and academic readiness. This could involve granting academic credit for approved wellness courses, considering student well-being in institutional strategic plans, and training faculty to support, rather than inadvertently hinder, student health efforts.

The pandemic case study (Li et al., 2024; Guo et al., 2025) proved that systemic disruption leads to systemic decline. Therefore, the solution must be equally systemic. By weaving together individual support, environmental redesign, and top-down policy, universities can transform from passive settings where health is a personal challenge into active health-promoting ecosystems. In such an environment, the healthy choice becomes the easy, normal, and valued choice, empowering all students not just the self-motivated to build and maintain a healthier lifestyle throughout their academic journey and beyond.

## 8 Limitations of the existing evidence

The conclusions advanced in this review must be qualified by several recurring limitations of the underlying literature. First, the predominance of cross-sectional designs constrains causal inference. Many of the most-cited studies on weight stigma, smartphone use, sleep, and BMI in university students (e.g., Saffari et al., 2022; Lu et al., 2025) report associations measured at a single time point and cannot determine whether stigma drives inactivity, inactivity drives stigma, or a third variable (e.g., depressive symptoms) drives both. Where longitudinal data are available, follow-up periods are typically short one academic semester to 3 years so true life-course inferences about whether university-period gains and losses persist into mid-adulthood remain extrapolative.

Second, measurement heterogeneity hampers cross-study synthesis. Self-reported physical activity, used in most cited surveys, is known to over-estimate actual activity, while accelerometer-based studies remain rarer in this population. BMI, the most common outcome, conceals body-composition changes that strength training and HIIT/SIT specifically target, and the recent identification of normal-weight obesity in approximately 22% of high-altitude student samples (Peng et al., 2025) suggests that BMI alone may misclassify metabolic risk in a substantial minority of normal-weight students. Psychological constructs are similarly heterogeneous: weight-related self-stigma, exercise self-efficacy, and motivational regulation are each measured by several non-equivalent scales, complicating direct comparison across studies.

Third, sample-size and replication issues warrant explicit acknowledgement. Several findings emphasized in this review rest on a single study and have not yet been independently replicated for example, the differential effect of early versus delayed time-restricted eating on weight loss versus anaerobic power (Yu and Ueda, 2025), and the predictive accuracy of the deep-learning BMI model reported by Mo et al. (2025). These are presented in this review as promising hypotheses rather than settled findings, and we use conditional language (“may,” “preliminary evidence suggests,” “in a single trial”) accordingly.

Fourth, the evidence base is mixed in at least two specific areas. (a) Aerobic versus high-intensity training for student weight loss: although Ma (2023) reported significant aerobic-induced weight loss, randomized head-to-head comparisons of HIIT/SIT versus moderate aerobic exercise within student samples are scarce and produce inconsistent superiority patterns; meta-analyses outside the student population suggest the two modalities yield comparable fat-mass changes when energy expenditure is matched, with adherence not biology often determining real-world superiority. (b) Direction of the activity–mental-health link: while several studies treat exercise as a buffer against anxiety and depression, the cross-sectional design of much of the cited work cannot rule out reverse causation (people with better mental health may exercise more).

Fifth, the literature is geographically and demographically uneven. A sizeable share of recent work originates from Chinese university samples (Lu et al., 2025; Li et al., 2024; Guo et al., 2025) and Saudi or Gulf samples (Alkilani et al., 2025; Mondal et al., 2025), with comparatively fewer recent contributions from Western European and North American universities. Generalizability across cultural contexts therefore requires caution, particularly for psychosocial constructs (weight-stigma norms, gender-specific body-image ideals) that are culturally patterned.

These caveats do not invalidate the integrative argument advanced in this review, but they bound it. Wherever a claim in the preceding sections rested on a single study, a non-replicated effect, or a culturally narrow sample, we have used conditional language, and the claim should be read as a hypothesis to be tested in larger, multinational, longitudinal, and methodologically rigorous future work.

## 9 Conclusion and future prospects

The challenge of weight reduction among university students cannot be understood or addressed through a singular lens. This comprehensive review elucidates that weight management in this population is a complex, dynamic interplay of physiological, psychological, and environmental factors. The evidence resoundingly argues against siloed solutions. A student’s journey toward a healthier weight is not merely a caloric equation but a process mediated by their self-perception, besieged by internalized stigma, challenged by academic stress, and competed with by digital seduction. Therefore, the most effective interventions are those that refuse to isolate physical exercise from the psychological and contextual fabric of student life. The cornerstone of a new paradigm is integration. Physiologically, this means moving beyond a sole reliance on traditional aerobic exercise to embrace a synergistic blend of modalities: the metabolic efficiency and time-respecting nature of High-Intensity and Sprint Interval Training (HIIT/SIT), the foundational cardiometabolic benefits of aerobic conditioning, and the crucial role of Muscle-Strengthening Exercise (MSE) in preserving metabolic rate and functional resilience. Psychologically, integration mandates that exercise programs be coupled with strategies to dismantle barriers: cognitive-behavioral techniques to combat weight stigma and build self-efficacy, motivational framing that links activity to stress relief and academic fortitude, and support for mental health to break the cycles of anxiety and poor sleep that derail healthy habits. Furthermore, true integration must be systemic. The university itself must be reconceptualized from a passive backdrop to an active, health-promoting ecosystem. This requires action at all levels: individual (through tailored, psychologically-informed programming), environmental (by creating active campuses and inclusive spaces), and policy-driven (by embedding wellness into curriculum, dining, and academic standards). Innovative tools, from technology-mediated platforms for scalable education and support to predictive analytics for early intervention, offer powerful means to personalize and scale these efforts. Ultimately, the goal must expand from short-term weight loss to the cultivation of Physical Literacy equipping students with the enduring motivation, competence, knowledge, and confidence to steward their own health. The transition to university is a formative period that sets trajectories for adult life. By implementing integrated, compassionate, and system-wide strategies, universities have a profound opportunity and responsibility. They can transform this high-risk period for weight gain into a launchpad for lifelong well-being, empowering students to build not only academic knowledge but also a resilient, healthy, and capable body and mind. The integration of fitness and psychology is not merely an academic exercise; it is an essential blueprint for fostering a healthier generation.

## 10 Future prospects

- Personalized digital health ecosystems: Develop and test integrated mobile platforms that combine real-time activity tracking, AI-driven personalized workout and nutrition coaching, cognitive-behavioral therapy (CBT) modules for mental health, and social connectivity features, all tailored to the university student’s schedule and stressors.
- Advanced predictive analytics and early intervention: expand the use of machine learning models beyond BMI prediction. Future systems could analyze combined datasets from fitness tests, campus card swipes (dining), and anonymized digital activity to predict risks for mental health crises, dropout due to health reasons, or the development of chronic conditions, triggering proactive, personalized support.
- Synergistic protocol research: Conduct rigorous clinical trials to explore the combined effects of specific dietary patterns (e.g., Time-Restricted Eating, protein-focused diets) with different exercise modalities (HIIT vs. steady-state aerobic) on body composition, metabolic health, and psychological outcomes in the student population to establish optimized, synergistic protocols.
- Scalable physical literacy curricula: Design, implement, and evaluate mandatory or incentivized university-wide “Physical Literacy” courses for first-year students. These would focus on building movement competence, exercise science knowledge, behavior-change skills, and positive body image, aiming to create a foundational shift in health mindset.
- Institutional “nudge” architecture and policy: Research the effectiveness of systemic environmental changes, such as dynamic classroom furniture, campus-wide “active break” alerts, gamified stairwell use, default healthy food options in dining halls, and policies linking physical activity to academic credit, to determine the most impactful ways to make healthy choices the effortless norm.
- Longitudinal and life-course studies: Establish long-term cohort studies that track students from enrollment through graduation and into early career, examining how university-based health interventions influence not only weight and fitness but also long-term healthcare utilization, career productivity, and overall quality of life.
- Equity-focused intervention design: Prioritize research that identifies and dismantles barriers to health resources for marginalized student groups. Future interventions must be co-designed with diverse populations to ensure accessibility, cultural relevance, and effectiveness in closing health disparity gaps on campus.
- Integration of telehealth and on-demand services: Explore hybrid care models that seamlessly blend in-person counseling, nutritionist visits, and fitness training with on-demand telehealth and digital support, providing continuous, flexible care that adapts to the variable and demanding student lifestyle.

## References

[ref1] Ali H. I. Attlee A. Alhebshi S. Elmi F. Al Dhaheri A. S. Stojanovska L. . (2021). Feasibility study of a newly developed technology-mediated lifestyle intervention for overweight and obese young adults. Nutrients 13:2547. doi: 10.3390/nu13082547, 34444707 PMC8399959

[ref2] Alkilani L. F. Z. Awad S. S. Altamimi J. Z. Alharbi F. S. (2025). Associations of nutritional awareness, body mass index, mental health, and fitness components among undergraduate university students. Front. Nutr. 12:1614296. doi: 10.3389/fnut.2025.1614296, 41070009 PMC12507332

[ref3] American College Health Association (2023). American College Health Association-National College Health Assessment III: Reference Group Executive Summary Fall 2022. Silver Spring, MD: ACHA.

[ref4] Ashari R. S. Sabirin R. M. Pratiwi D. A. Tsania M. N. Merlinda S. T. B. Wibowo R. A. (2025). Translation, adaptation and measurement properties of the muscle-strengthening exercise questionnaire among university students in Indonesia. BMJ Open 15:e102211. doi: 10.1136/bmjopen-2025-102211, 40897480 PMC12406860

[ref5] Bandrés S. M. Conty J. L. M. Polonio-Lopez B. Diaz-Gonzalez S. Rivera-Picon C. Rodríguez-Cañamero S. . (2025). Weight gain and fatigue effect on nursing students performing high-fidelity CPR simulation. J. Clin. Med. 14:7483. doi: 10.3390/jcm14217483, 41226879 PMC12608659

[ref6] Bandura A. Freeman W. H. Lightsey R. (1999). Self-efficacy: the exercise of control. J. Cogn. Psychother., 13, 158–166. doi: 10.1891/0889-8391.13.2.158

[ref8] Blanco-Ferreiro A. Candal-Pedreira C. Sendón B. Santiago-Pérez M. I. Rey-Brandariz J. Varela-Lema L. . (2024). Self-perceived body weight and weight status: analysis of concordance by age group and sex. Public Health 229, 160–166. doi: 10.1016/j.puhe.2024.02.007, 38447299

[ref9] Blouin J. Feek A. Jin Y. Cook J. O'Neal T. Sacheck J. M. (2025). The fitness, rest, and exercise for strength and health (FRESH) study: a three-year comparison of college students' perceived and measured health metrics. Nutrients 17:217. doi: 10.3390/nu17020217, 39861347 PMC11767257

[ref10] Bray S. R. Born H. A. (2004). Transition to university and vigorous physical activity: implications for health and psychological well-being. J. Am. Coll. Heal. 52, 181–188. doi: 10.3200/JACH.52.4.181-188, 15018429

[ref11] Bray G. A. Frühbeck G. Ryan D. H. Wilding J. P. (2016). Management of obesity. Lancet 387, 1947–1956. doi: 10.1016/S0140-6736(16)00271-3 26868660

[ref12] Cairney J. Dudley D. Kwan M. Bulten R. Kriellaars D. (2019). Physical literacy, physical activity and health: toward an evidence-informed conceptual model. Sports Med. 49, 371–383. doi: 10.1007/s40279-019-01063-3, 30747375

[ref13] Chen B. Cao R. Pan L. Song D. Liao C. Li Y. (2024). Association among physical activity, anxiety and oral health status in Chinese university students: a cross-sectional study. Heliyon 10:e24529. doi: 10.1016/j.heliyon.2024.e24529, 38312590 PMC10835240

[ref14] Chwałczyńska A. Andrzejewski W. (2021). Changes in body mass and composition of the body as well as physical activity and time spent in front of the monitor by students of the Wroclaw University of Health and Sport Sciences during the period of COVID-19 restrictions. Int. J. Environ. Res. Public Health 18:7801. doi: 10.3390/ijerph18157801, 34360094 PMC8345687

[ref15] Deci E. L. Ryan R. M. (2000). The “what” and “why” of goal pursuits: human needs and the self-determination of behavior. Psychol. Inq. 11, 227–268. doi: 10.1207/S15327965PLI1104_01

[ref16] Deforche B. Van Dyck D. Deliens T. De Bourdeaudhuij I. (2015). Changes in weight, physical activity, sedentary behaviour and dietary intake during the transition to higher education: a prospective study. Int. J. Behav. Nutr. Phys. Act. 12, 1–9. doi: 10.1186/s12966-015-0173-9, 25881147 PMC4332914

[ref21] Greenhalgh T. Thorne S. Malterud K. (2018). Time to challenge the spurious hierarchy of systematic over narrative reviews? Eur. J. Clin. Investig. 48:e12931. doi: 10.1111/eci.12931, 29578574 PMC6001568

[ref22] Guo L. Jiang L. Huang H. (2025). A longitudinal study of four-year changes in physical fitness among university students before and after COVID-19: 2019-2022. PLoS One 20:e0334088. doi: 10.1371/journal.pone.0334088, 41071790 PMC12513638

[ref23] Huang G. Chen Y. Lee B. Qiu Y. Mao A. Liang M. . (2025). A study on the effects of modified sprint interval training on physical fitness test scores and the quantitative and dose-response relationships among Chinese male university students. Front. Physiol. 16:1555019. doi: 10.3389/fphys.2025.1555019, 40070460 PMC11893556

[ref25] Kilpatrick M. Hebert E. Bartholomew J. (2005). College students' motivation for physical activity: differentiating men's and women's motives for sport participation and exercise. J. Am. Coll. Heal. 54, 87–94. doi: 10.3200/JACH.54.2.87-94, 16255320

[ref27] Kwan M. Y. Cairney J. Faulkner G. E. Pullenayegum E. E. (2012). Physical activity and other health-risk behaviors during the transition into early adulthood: a longitudinal cohort study. Am. J. Prev. Med. 42, 14–20. doi: 10.1016/j.amepre.2011.08.026, 22176841

[ref28] Kwilosz E. Musijowska M. Badora-Musial K. Zadarko E. Zadarko-Domaradzka M. (2025). Eating habits, physical activity, body composition and cardiorespiratory fitness in university students: a cross-sectional study. Nutrients 17:3166. doi: 10.3390/nu17193166, 41097243 PMC12526070

[ref30] Li H. Song Y. Wang Y. Feng X. Li C. Peng J. . (2024). Impact of the COVID-19 pandemic lockdown on body mass index: a three-year follow up study in 6,156 Chinese college students. Front. Endocrinol. 15:1387151. doi: 10.3389/fendo.2024.1387151, 38966211 PMC11222588

[ref31] Liu Y. Liu H. Liu Z. (2021). The relationship of self-presentation, psychological needs, and exercise dependence in college students with overweight. Front. Psychol. 11:625501. doi: 10.3389/fpsyg.2020.625501, 33551935 PMC7862777

[ref34] Lu Y. Tian H. Shi W. Liu H. Wu J. Tao Y. . (2025). Associations between mobile phone involvement, BMI levels, and sleep quality among Chinese university students: evidence from a multi-regional large-scale survey. Front. Public Health 13:1533613. doi: 10.3389/fpubh.2025.1533613, 40034171 PMC11872715

[ref36] Ma J. (2023). Effects of aerobic exercise on body morphology in obese university students. Rev. Bras. Med. Esporte 29:e2022_0221. doi: 10.1590/1517-8692202329012022_0221

[ref38] Mo M. Luo W. Hu Q. Wang J. Jiao T. Xie L. . (2025). A deep hybrid learning framework with attention-enhanced feature extraction for BMI prediction based on physical fitness. Front. Public Health 13:1640226. doi: 10.3389/fpubh.2025.1640226, 40963657 PMC12436491

[ref40] Mondal S. Basu C. Alkhawaitri M. Almamari I. Albrwaney S. Alhabsi T. (2025). Obesity among college students in Oman: implications for health and academic performance. BMC Public Health 25:1111. doi: 10.1186/s12889-025-21946-7, 40128737 PMC11931841

[ref41] Nelson M. C. Story M. Larson N. I. Neumark-Sztainer D. Lytle L. A. (2008). Emerging adulthood and college-aged youth: an overlooked age for weight-related behavior change. Obesity 16, 2205–2211. doi: 10.1038/oby.2008.365, 18719665

[ref43] Peng N. Li J. Liu B. Yan X. Ouzhu N. (2025). Normal-weight obesity in high-altitude youth: gender disparities and protective effects of native adaptation. PLoS One 20:e0328992. doi: 10.1371/journal.pone.0328992, 40748963 PMC12316246

[ref44] Rauff E. L. Kumazawa M. (2024). Physical activity motives and self-efficacy to overcome physical activity barriers in first-year undergraduates: do they differ based on physical activity levels? J. Am. Coll. Heal. 72, 2242–2249. doi: 10.1080/07448481.2022.2109032, 35943966

[ref45] Ryan R. M. Deci E. L. (2017). Self-Determination Theory: Basic Psychological Needs in Motivation, Development, and Wellness. New York, NY: Guilford Press. Revue québécoise de psychologie 38, 231–234. doi: 10.7202/1041847ar

[ref46] Saffari M. Chen J. Wu H. Fung X. Chang C. Chang Y. . (2022). Effects of weight-related self-stigma and smartphone addiction on female university students' physical activity levels. Int. J. Environ. Res. Public Health 19:2631. doi: 10.3390/ijerph19052631, 35270328 PMC8909679

[ref47] Scurt M. D. Balint L. Mijaica R. (2022). Improving body mass index in students with excess weight through a physical activity programme. Children 9:1638. doi: 10.3390/children9111638, 36360366 PMC9688828

[ref49] Sisay T. (2021). Physical inactivity as a pandemic: daily activities and dietary practices. Risk Manage Healthcare Policy 14, 3287–3293. doi: 10.2147/RMHP.S317440, 34408514 PMC8364389

[ref50] Steinberg L. D. (2014). Age of Opportunity: Lessons from the New Science of Adolescence. Boston, MA: Houghton Mifflin Harcourt. 264 pp. Available online at: https://books.google.com/books?hl=zh-CN&lr=&id=sppdBAAAQBAJ&oi=fnd&pg=PP1&dq=Steinberg,+L.+(2014).+Age+of+Opportunity:+Lessons+from+the+New+Science+of+Adolescence.+Houghton+Mifflin+Harcourt.&ots=Adv5_cp2yO&sig=1ehTaPNBf9C5iAsaAjW4hGZfKLE

[ref51] Sukhera J. (2022). Narrative reviews: flexible, rigorous, and practical. J. Grad. Med. Educ. 14, 414–417. doi: 10.4300/JGME-D-22-00480.1, 35991099 PMC9380636

[ref52] Tan L. Kwanmuangwanic P. (2025). Effects of tennis exercise of different intensities on cardiorespiratory capacity and mental health of sedentary students. J. Sports Med. Phys. Fitness 65, 1286–1295. doi: 10.23736/S0022-4707.25.16710-8, 40673786

[ref53] Tang H. Wang J. Bao J. Zhang L. (2024). Physical fitness decline and career paths: a longitudinal study of medical undergraduates. BMC Med. Educ. 24:513. doi: 10.1186/s12909-024-05493-0, 38720325 PMC11080080

[ref54] Teixeira P. J. Carraça E. V. Markland D. Silva M. N. Ryan R. M. (2012). Exercise, physical activity, and self-determination theory: a systematic review. Int. J. Behav. Nutr. Phys. Act. 9:78. doi: 10.1186/1479-5868-9-78, 22726453 PMC3441783

[ref55] Tomiyama A. J. (2019). Stress and obesity. Annu. Rev. Psychol. 70, 703–718. doi: 10.1146/annurev-psych-010418-102936, 29927688

[ref57] Vartanian L. R. Porter A. M. (2016). Weight stigma and eating behavior: a review of the literature. Appetite 102, 3–14. doi: 10.1016/j.appet.2016.01.034, 26829371

[ref58] Whitehead M. (Ed.). (2010). Physical Literacy: Throughout the Lifecourse. Routledge. doi: 10.4324/9780203881903

[ref61] Wynn K. B. Severt B. Mann D. (2025). Annual fitness testing of exercise science students: a descriptive study. Quest, 1–19. doi: 10.1080/00336297.2025.2595042

[ref62] Yan W. Nie M. Ma R. Guo Q. Li H. (2024). The influence of physical literacy of student with different obesity levels on physical fitness: the mediating effect of MVPA. Front. Public Health 12:1463108. doi: 10.3389/fpubh.2024.1463108, 39430712 PMC11486707

[ref63] Yu Z. Ueda T. (2025). Time-restricted eating without exercise enhances anaerobic power and reduces body weight: a randomized crossover trial in untrained adults. Nutrients 17:3011. doi: 10.3390/nu17183011, 41010536 PMC12473138

